# Cost evaluation model to compare in house repackaging, repackaging vendors, and sourcing unit dose medications from manufacturers for oral liquids

**DOI:** 10.1016/j.rcsop.2022.100157

**Published:** 2022-07-13

**Authors:** Matthew Kelm

**Affiliations:** ASHP Global Consulting Services**,** 4500 East-West Highway, Suite 900, Bethesda, MD 20814, United States of America

**Keywords:** Cost, Packaging, Repackaged, Decision, Analysis, Technology

## Abstract

**Purpose:**

Unit-dose packaging systems are widely used and accepted practices in many hospitals in the US. When adopting a unit-dose, there are three different avenues in which pharmaceuticals can be obtained. Products can be purchased from a manufacturer-produced source, outsourced to repackaging by a 3rd-party repackaging service or repackaged in-house by investing in the technology and the resources to do so. Prior literature has suggested that manufacturer-based unit-dose purchasing was associated with a 1% cost savings over repackaged unit-dose. In this study, we hope to take a more extensive look at the cost and concerns associated specifically with unit dose liquids when purchased from a manufacturer, outsourced to a third party repackager, or repackaged from bulk bottles with in-house technology and resources.

**Methods:**

A cost evaluation model, which factored in cost associated with used and expired product, was utilized to estimate and compare the cost of the three systems.

**Results:**

Overall cost between the three systems was largely similar, although manufacturer-based repackaging was determined to be the most cost effective system.

**Conclusion:**

The results of this decision model analysis suggests that the cost associated with purchasing unit dose liquids from manufacturers, third party repackagers, and in-house repackaging are similar. Therefore, utilizing a specific system is unlikely to make a significant impact on the overall pharmaceutical budget for a large hospital or health system.

## 1 Introduction

Medication errors are a known risk in every phase of the medication use process, but literature demonstrates that errors occur most frequently in the prescribing and administration stages.^1^ Since its introduction in the 1960s, unit-dose packaging systems have gained wide acceptance as the standard of practice for dispensing medications in hospitals in the US.^2^ By pairing unit-dose packaging with barcode medication administration practices, reductions in the error rates at the point of administration are observed.^3^ In article by Meller and colleagues,^4^ a commentary on the packaging options is presented. While they addressed the oral solid market, they did not address the oral liquid market nor the challenges present with three liquid packaging options to decide from. Liquid packaging is of particular interest due to the variance in pricing of bulk bottle vs. unit dose cups and the investment needed for liquid packing automation. When deciding to adopt a unit-dose, barcode-encoded dispensing and administration infrastructure, pharmacy leaders have three primary mechanisms to achieve this standard. Products can be purchased from a manufacturer-produced source, outsourced to repackaging by a 3rd party repackaging service or repackaged in-house by investing in the technology and the resources to do so.^16^, 17. Literature has been published comparing the costs of manufacturer unit-dose oral solids to in-house repackaged unit dose in long term care patient populations. In an analysis by Stephenson and colleagues,^5^ manufacturer-based unit-dose purchasing was associated with a 1% cost savings over repackaged unit-dose solids. Additionally, this study conducted a sensitivity analysis that indicated when factoring in the total cost of production, waste, reduction in beyond use date and product loss, as long as the acquisition cost of unit dose drugs was no >5.1% higher than the cost of a bulk bottle, manufacturer-based unit-dose will be less expensive.

Cost evaluation models provide a framework for compiling clinical and economic evidence in a systematic fashion. In healthcare, this model is best suited for situations in which one must choose between two or more options where there may be meaningful tradeoffs between alternative strategies. One of the most significant limitations of Stephenson and colleagues' work^5^ was that the results were based on the cost and utilization at one long-term care pharmacy. Additionally, in their review, they did not include an analysis of third-party repackaging services which can limit the use of this data in larger hospitals in which these services may be utilized. In this study, we compared unit dose liquids when purchased from a manufacturer, outsourced to a third party repackager, or repackaged from bulk bottles with in-house technology and resources. Overall, the following research seeks to build upon prior knowledge of oral solid repackaging and will take a more extensive look at the cost, unique complexities for liquid formulations, and concerns associated with packaging systems in a hospital environment over a 6-month period. Specifically, this research provides a financial analysis model detailing considerations for three distinct repackaging methodologies and highlights nuances specific to liquid unit doses.

## 2 Methods

### 2.1 Data and timeframe determination

**Fig. 1:**
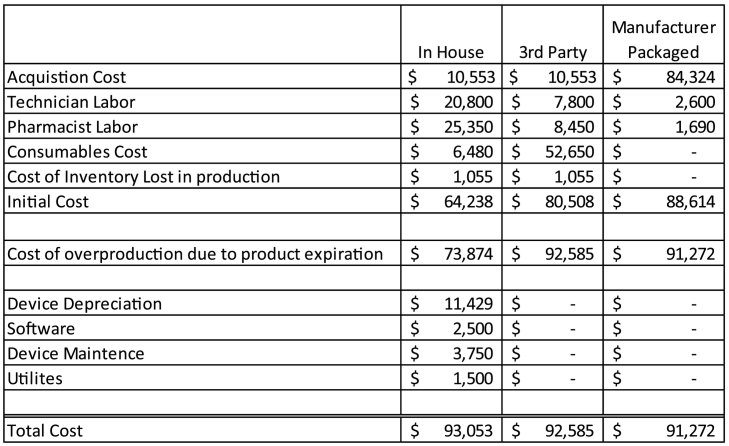
Results of Decision Analysis Model for Comparing Cost of Manufacturer, 3rd Party and In-house Repackaged liquids over a six-month period. Calculation Methodology (In-house example): (Acquisition Cost of Drugs + Technician Labor + Pharmacist Labor + Repackaging Consumables (total doses x cost per dose) x 1.1 to account for production needed for lost inventory in packaging process. The sum of these figures are labeled initial cost. Initial cost is then multiplied by the percent of over production required due to expiration. This value is then combined with 6 months device depreciation +6 month software license +6 month device maintenance +6 month device utilities to determine the total cost of the operation.

Similar to Pazour and colleagues,^6^ a cost evaluation model was used to estimate and compare the costs of manufacturer, third party repackaged, and in-house repackaged unit dose systems. Costs associated with product production and expiration were totaled for the three sources and a comparison was established (Fig. 1**)**. Acquisition costs for the model were obtained through wholesaler supplied acquisition cost (WAC) for seven high volume oral medications. The seven selected agents were: (1) Acetaminophen 650 mg/20.3 mL; (2) Acetaminophen 160 mg/5 mL; (3) Ibuprofen 100 mg/5 mL; (4) Docusate Sodium 100 mg/10 mL; (5) Guaifenesin 200 mg/10 mL; (6) Lactulose 20 g/30 mL, and (7) Valproic Acid 250 mg/5 mL. These line items were selected due to their volume of use in the hospital and availability from a commercial manufacturing source. Purchasing volume was determined by retrieving 6 months of purchase history data between June 2020 and November 2020 from a 1000 bed academic medical center. Additional cost assumptions are detailed in Table 1 including staffing, repackaging, and destruction (of outdated medication) cost. An important aspect to highlight in the third party repackaged and in-house repackaged models is product lost in the packing process or yield. For example when packaging a 480 mL bottle into 10 mL cups, there is a 10% loss of inventory from line priming, incompletely filled or sealed cups, etc.

**Table 1:** Sources of data⁎

Type of Data	Source of Information	In-house Repacked	3rd Party Repackaged	Manufacturer Packaged
Acquisition Cost of Drugs (6 months)	Wholesaler Purchase Data	$10,553	$10,553	$84,324
Percent of Inventory loss in packing process (ie line priming and incompletely filled cups)	Observation from repacking process and yield from bulk liquid	10%	10%	N/A
Percent of Inventory loss due to expiration	Hospital Reverse Distributor Data	15%	15%	3%
Technician Labor Expense	Pharmacy Administrator Experience	$20/h inclusive of benefits 8 h, 5 days weekly: **$20,800/ 6 months**	$20/h inclusive of benefits 3 h, 5 days weekly **$7800 / 6 months**	$20/h inclusive of benefits 1 h, 5 days weekly **$2600 / 6 months**
Pharmacist Labor Expense	Pharmacy Administrator Experience	$65/h inclusive of benefits 3 h, 5 days weekly **$25,350/ 6 months**	$65/h inclusive of benefits 2.5 h, 2 days weekly **$8450 / 6 months**	$65/h inclusive of benefits 0.5 h, 2 days weekly **$1690 / 6 months**
Repacking consumables cost	Pharmacy purchasing data/Vendor Price Quotes	$0.08 per dose **$6480 / 6 months**	$0.65 per dose $**52,650 / 6 months**	N/A
Repacking Device (depreciated over 7 years)	Pharmacy Purchasing Data	$160,000	N/A	N/A
Repacking Software License (6 months)	Pharmacy Purchasing Data	$2500	N/A	N/A
Repacking device maintenance (6 months)	Repacking Purchase Agreement	$3750	N/A	N/A
Utilities and other expenses to run repackager (6 months)	Pharmacy Administrator Experience	$1500	N/A	N/A

⁎ Assumptions from staffing, salary, and benefits estimates are median values and were determined through review of personnel salary data.

### 2.2 Labor component

The labor expenses in this analysis require additional detail. For the in-house repackaged analysis, technician labor includes daily device set up, inventory assessment, packaging time, device cleaning and maintenance, supply ordering, storage of final packaged product and lot documentation. Pharmacist labor associated with in-house packing includes technician training, database record validation for compounding records, individual review of lot numbers, supervisory oversight of the packaging technician, policy and procedure development, and quality analysis of packaging operations. The percentage product loss due to expiration was determined through review of reverse distributor data at the NDC level on expired pharmaceuticals removed from the facility over a 6-month period. For the in-house packaging option, the cost of the automation to support repackaging is depreciated over a period of seven years to estimate the useful life of the equipment. For the 3rd party repackaged labor, technician labor expenses included inventory review, supply coordination with 3rd party, receipt of repackaged doses, reconciliation of inventory purchased doses received, storage of final packaged product, and coordination with invoices and accounting. Pharmacist labor associated with 3rd party repackaging includes vendor relationship management, supervisory oversight of the pharmacy technician, product quality assurance, purchasing optimization assessments, and shortage and substitution management. Lastly, when working with a third party repackager, a buyer will need to factor in turnaround time from shipment of bulk product to the facility, time for repackaging, and return of inventory to the hospital. Literature has estimated turnaround time to vary from three to ten days.^7^ The turnaround times should be specified in the agreement with the vendor. A pharmacy buyer will need to account for this turnaround time in determining inventory levels for repackaging. For the manufacturer packaged model, technician labor costs would be inclusive of inventory review, order placement and order receipt functions. The pharmacist labor costs would include supervisory oversight of the pharmacy technician, purchasing optimization assessments, and shortage and substitution management.

### 2.3 Sensitivity analysis

Additionally, a sensitivity analysis was performed to quantify the impact of assumptions for percent of inventory lost due to expiration. Calculation methodology remained consistent, but percent of inventory loss due to expiration was varied for in-house and repackaged products from 3%, 5%, 10%, 15% and 20%. Manufacturer expiration was held constant at 3%.

## 3 Sources of data

Purchasing volume data over a 6 month period from a pharmaceutical distributor was used to identify the seven high volume drugs used in this study. The data generated from these seven agents included a total of 81,000 unit dose cups. Assumptions from staffing, salary, and benefits estimates are median values and were determined through review of personnel salary data from the author's institutional experience. The percentage product loss due to expiration was determined through review of reverse distributor data on expired pharmaceuticals removed from the facility over a 6-month period.

## 4 Results

**Fig. 2:**
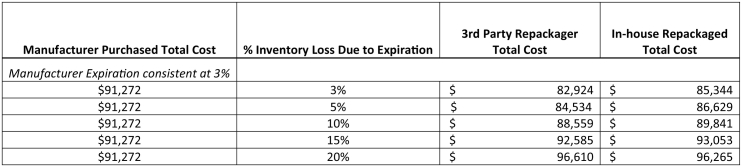
Sensitivity Analysis for Changes in Expiration Percentage Comparing Cost of Manufacturer, 3rd Party and In-house Repackaged liquids over a six-month period.

Fig. 1 demonstrates the outcome of this analysis. Overall, a manufacturer-based model resulted in costs of $91,272, while in-house packaging was calculated $93,053, and third party repackaging resulted in a total cost of $92,585. These results show that costs associated with any of the three models are similar. Fig. 2 details the outcomes of the sensitivity analysis. By varying the input assumptions on product expiration, differences may be realized in the outcome. Specifically, as inventory loss due to expiration increases, total cost increases.

## 5 Discussion

This paper provides a framework for a pharmacy administrator to incorporate the economic impact of their decision given that no current published literature addresses oral liquid packaging. Overall, manufacturer repackaging was determined to be the most cost effective system, although cost between the three systems was largely similar. When comparing the most expensive to the least expensive system, there is a minimal cost difference of $1781 (1.9%). In the context of the larger pharmaceutical budget for a pharmacy department, the difference amongst these systems is relatively minor. The cost evaluation model described in this report is a useful framework for evaluating the costs associated with three models of providing unit dose oral liquids. In addition to considering cost, the benefit of convenience should also be considered. Institution-specific factors such as physical space, competing labor priorities, and administrative capacity to conduct the needed analysis are factors likely to be decisive in determining the unit-dose for oral medications model a hospital or health system may pursue. As demonstrated in Table 1, the work hours associated with in-house repackaging would require dedicated resources of approximately 0.5 FTE pharmacist and 1.2 FTE pharmacy technician, when factoring in leave and associated backfill costs. On the other hand, the labor needed to support a manufacturer-based model could likely be incorporated into the job responsibilities of existing staff members. Factors such as the labor estimate used and assumptions on product loss due to expiration were determinate factors in the outcome. A limitation of the labor assumption is that it represents a single center in one geographic location of the United States. Regional differences may alter the labor cost calculation. As demonstrated in Fig. 2, loss due to expiration results in additional product needing to be packaged to make up for the loss. If a heath-system chooses to pursue the in-house repackaging option, important precautions and regulatory considerations should be considered. ASHP has published a Technical Assistance Bulletin on repackaging oral solids and liquids in single unit and unit-dose packages.^15^ In this bulletin they outline 15 specific guidelines for the procedures for repackaging products in a safe manner. These guidelines span facility recommendations, environmental conditions, procedural steps, quality assurance activities, control records, and storage.^8^

Minimum standards for unit-dose repackaging have been established in several United States Pharmacopeia (USP) Chapters. Specifically, chapters 9–13 (inclusive) provide information on the repackaging of oral liquids include:9., 10., 12., 13.

• Chapter <7> Labeling
• Chapter <681> Repackaging into Single-Unit Containers and Unit-Dose Containers for Nonsterile Solid and Liquid Dosage Forms.
• Chapter <795> Pharmaceutical Compounding—Nonsterile Preparations
• Chapter <1136> Packaging- Unit-of-Use
• Chapter <1178> Good Repackaging Practices

In-house based repackaging programs offer a department the greatest flexibility in product production, formulation, and can be useful in navigating product shortages in the market. An in-house packing model allows a department maximum flexibility in the production volume and presentation of unit doses. By developing both capacity and capability a department can overcome challenges encountered by drug shortages or needs for alternative formulations. An important distinction is needed between unit dose liquid volume and individually prepared oral/enteral syringes. Unit dose liquids, often provided in cups are useful when dispensing liquids in a fixed volume that will easily satisfy the standard dose of a medication, for example 325 mg of acetaminophen. This formulation is distinct from the patient specific, customized dose pharmacies frequently prepare for pediatric patients or when the prescribed dose is not standardized.

Limitations are present in this analysis. In regards to acquisition cost used in this study, it is important to note that actual costs of commercially available unit dose cups are typically significantly below WAC. Group Purchasing Organization (GPO) contractor price plus wholesaler fees may show greater parity. The difference from WAC price to GPO price can vary from 17% to 72%. The average difference between WAC and GPO is 46%. GPO price was not able to be used in this analysis, due to confidentiality of those contractual arrangements. As stated in the results, minor adjustments to the inputs for labor costs, product expiration, or other variables, may result in different outcomes. Additionally, factors such as rebates, or other contractual incentives are not reflected. The analysis does not take into account items for which a commercial source is not available. For this reason, a pharmacy department may choose to retain limited repackaging capability. Lastly, the paper is limited in its ability to provide a comparison to other similar studies, as no published study was able to be found specifically addressing the nuances of oral liquid repackaging.

Another important consideration for a department to include in the evaluation of unit dose models include product quality. By purchasing unit dose liquids from a cGMP-compliant commercial packager, the highest quality standards of the industry are observed. This, along with the maximum beyond use dating, are notable advantages. As observed in the higher expiration rates for repackaged or in-house compounded formulations, USP chapter <795> states non-preserved aqueous dosage forms that are packaged in tight, light-resistant containers are limited to a 14 day beyond use date unless there is a specific stability study for longer dating.^11^ Both the Food and Drug Administration (FDA) and The Joint Commission recommend procurement of pharmaceutical manufacturers' packages when possible.^8^^,^^14^

## 6 Conclusion

The results of this cost evaluation analysis suggest that the cost associated with purchasing unit dose liquids from manufacturers, third party repackagers, and in-house repackaging are similar. Therefore, utilizing a specific system is unlikely to make a significant impact on the overall pharmaceutical budget for a large hospital or health system.

## Declaration of Competing Interest

None.
